# Effects of Postconditioning with Fructose on Arrhythmias and the Size of Infarct Caused by Global Ischemia and Reperfusion in Isolated Rat Heart

**DOI:** 10.15171/apb.2018.007

**Published:** 2018-03-18

**Authors:** Jila Haghi, Tahereh Eteraf-Oskouei, Moslem Najafi

**Affiliations:** ^1^Student Research Committee, Faculty of Pharmacy, Tabriz University of Medical Sciences, Tabriz, Iran.; ^2^Department of Pharmacology and Toxicology, Faculty of Pharmacy, Tabriz University of Medical Sciences, Tabriz, Iran.

**Keywords:** Fructose, Postconditioning, Ischemia/Reperfusion, Isolated heart

## Abstract

***Purpose:*** In the present study, postconditioning effect of fructose against ischemia/reperfusion (I/R)-induced arrhythmias and infarct size were investigated in isolated rat heart.

***Methods:*** The isolated hearts were divided into 7 groups, mounted on a Langendorff apparatus at constant pressure then subjected to 30 min zero flow global ischemia followed by 120 min reperfusion. In the control group, normal Krebs–Henseleit (K/H) solution was perfused into the hearts throughout the experiment. In two separate sets of experiments, the treatment groups received 12, 24 and 48 mM of fructose with/without normal glucose in K/H solution for 20 min at the beginning of reperfusion. Cardiac arrhythmias including number of ventricular tachycardia (VT), total ventricular ectopic beats, incidence and duration of VT, reversible and irreversible ventricular fibrillation were recorded and analyzed during the first 30 min of reperfusion. Computerized planimetry method was used to determine volume and percentage of infarct size.

***Results:*** Administration of fructose as a postconditioning agent clearly reduced volume and percentage of infarct size in the all treatment groups. The effect was statistically significant especially in the hearts that treated by fructose plus glucose (P<0.05). However, fructose alone or its co-administration with glucose had no significant inhibitory effect against reperfusion arrhythmias.

***Conclusion:*** The results showed that perfusion of high concentration of fructose alone or coincident with glucose in globally ischemic-reperfused isolated rat hearts can reduce infarct size without inhibitory effect against reperfusion arrhythmias. Probably, fructose by providing adequate ATP for cardiac functions may inhibit necrosis and death of cardiomyocytes during I/R.

## Introduction

Despite the major advances in the identification and treatment of cardiovascular diseases, myocardial infarction (MI) continues to be a major health problem.^1^ Other important disorders such as arrhythmias, are also created by MI.^2^ In this regard, size of the heart infarction area is an important determinant for mortality after MI.^3^ Restoring blood flow to ischemic myocardium leads to ischemia/reperfusion (I/R) injury.^4^ Improvement of cardiac function in reperfusion mainly depends on the duration of ischemia. On the other hand, reperfusion itself causes disorders that include myocardial stunning, re-flow phenomenon, cardiac arrhythmias, necrosis and apoptosis of cardiomyocytes.^5,6^

Fructose or Laevulose (C_6_H_12_O_6_) is a sugar with colorless or granular white and odorless crystalline, which is highly soluble in water.^7^ It is converted into glycogen in the liver and metabolized faster than dextrose without the need for insulin. Therefore, it can be used in diabetic patients, but it should not be used in hypoglycemia and people with congenital fructose intolerance.^7^ Natural sources of fructose include fruits, vegetables and honey. In equal amounts, fructose is sweeter than sucrose and glucose and is used today as a common sweetener.^8^ In the past, receiving fructose from food was approximately 16-20 g/day and mostly from natural sources, while over the past three decades, its consumption in the diet has increased to nearly 85-100 g/day due to consumption of industrial products such as carbonated beverages, industrial juices, canned fruits, jams and jellies.^9^

Myocardium can be susceptible to the harmful effects of long-term fructose nutrition, because the heart tissue is sensitive to insulin and dependent on glycolysis.^10^ Studies on rats have shown that feeding with high levels of fructose increases blood pressure. One of the possible mechanisms associated with this effect of fructose is insulin resistance, leading to hyperinsulinism, which increases the blood pressure by stimulating activity of the sympathetic nervous system and also reabsorption of sodium from the kidneys.^11^ Moreover, high-fructose-fed rats have left ventricular hypertrophy, which appears to be associated with increased activity of the renin-angiotensin system and stimulation of sympathetic activity.^12^

Although numerous studies on rodents have shown increased fructose-induced hypertension,^13,14^ in most studies of acute fructose administration in humans, there was no increase in the activity of the sympathetic system followed by increased blood pressure.^15^ Also, fructose feeding in rats caused fibrosis around the coronary arteries, which was associated with an increase in Interleukin-18. Arterial vascular fibrosis is one of the most prominent pathologic changes in patients with myocardial ischemia.^16^ Fructose also causes dysfunction of end arterioles in skeletal muscles^17^ and endothelial dysfunction due to inhibition of nitric oxide synthesis.^18^ Other studies have also shown that fructose nutrition facilitates oxidative destructive effects by interfering with antioxidant defense and exacerbating the production of free radicals.^19^ In addition, some studies have suggested that increasing fructose consumption and increase in the prevalence of obesity, type 2 diabetes and metabolic syndrome are in parallel and worrisome.^20^ However, there are no clear evidences based on epidemiologic studies on the relationship between the intake of fructose in normal diets and the risk factors for cardiovascular diseases.^21^ Therefore, some studies show the harmful effects^22^ and others the protective role for fructose in the damage caused by myocardial reperfusion.^23^ Results of our previous study demonstrated that preconditioning with fructose (its administration prior to ischemia) reduces the size of infarcted area and arrhythmias caused by I/R phenomenon.^24^

To the best of our knowledge, postconditioning effects of fructose against I/R-induced arrhythmias and infarct size has not previously been studied. Hence, due to the contradictory results of previous studies and to better understand the effects of fructose on the heart in I/R conditions, postconditioning effects of fructose on the size of infarct and arrhythmias caused by global ischemia and reperfusion in the rat’s isolated heart were investigated in this study.

## Materials and Methods

### Animals

Male Wistar albino rats weighing 250 to 280 g were kept in seven-member groups in standard transparent polyethylene cages in a light cycle (12 h of light - 12 h of darkness) in an animal room at Tabriz Faculty of Pharmacy at 23±2 °C. They had free access to water and food until the time of test.

### Preparation of Krebs-Henseleit solution

Krebs-Henseleit (K/H) solution was freshly prepared before each test. The components of the solution (in mmol/ l) included sodium chloride (118.5), sodium bicarbonate (25), potassium chloride (4.8), magnesium sulfate (1.2), D-glucose (12), potassium dihydrogen phosphate (1.2), and calcium chloride (1.7). All of these ingredients were dissolved in distilled water and, to prevent precipitation, calcium chloride was added after all the ingredients.^25,26^

### Surgical procedure

The rats were divided into seven groups randomly as a control and six treated groups (n=7-10 in each group). The animals were pretreated with intraperitoneal (i.p.) injection of 300 IU heparin then anaesthetized by sodium pentobarbital (50-60 mg/kg, i.p.).^26,27^ After sacrifice of the animals, their hearts were excised rapidly and mounted on a non-recirculating Langendorff apparatus under constant pressure of 100 mmHg at 37 °C and perfused with K/H solution that previously equilibrated with 95% O_2_-5% CO_2_ (pH=7.4).^28^ A latex fluid filled balloon was inserted into the left ventricle and inflated to give a preload of 8-10 mmHg.^29^

### Experimental protocols

After 25 min stabilization period, the isolated hearts were subjected to 30 min zero flow global ischemia followed by 120 min reperfusion. In the control group, the hearts were perfused by normal K/H solution throughout the experiment. In the first set of treatment groups, the hearts received glucose-free K/H solution in which fructose replaced glucose at concentrations of 12, 24 and 48 mM for 20 min at the beginning of reperfusion time. In the another set of treatment groups, the hearts were perfused with fructose at 12, 24 and 48 mM coincident with glucose in K/H solution for 20 min at the beginning of 120 min reperfusion then continued perfusion with normal K/H solution. During the experiment, an epicardial ECG was recorded continuously by a physiograph (Narco, MR–III–S, USA).

### Arrhythmias analysis

Based on the Lambeth conventions, the ECGs were analyzed to determine the number of single ectopic beats, salvos (couplets and triplets), ventricular tachycardia (VT), the total number of ventricular ectopic beats (VEBs), incidence and duration of VT and reversible ventricular fibrillation (Rev VF), and incidence of irreversible VF (Irrev VF) during the first 30 min of reperfusion time.^30^

### Measuring infarct size

After 120 min reperfusion, the isolated hearts were frozen at -20 °C then cut into pieces with 1-2 mm thick transverse sections. The pieces of each heart were incubated in a test tube containing 5 ml of 1% triphenyltetrazolium chloride in phosphate buffer for 15 min at 37 °C.^31^ After that, the tissue pieces were fixed in 10% formalin solution for 24 h, and after drying with filter paper, they were placed on a thin piece of glass. The second piece of glass was placed on the pieces of heart and the two glass sheets were tightened together. Subsequently, a transparent paper was placed on the glass and both infarcted and non-infarcted areas were marked. Finally, the infarct size was measured by computerized planimetric method and Land Calc software.^31^

### Statistical Analysis

Except for the percentage of occurrence of VT and VF, the other data are expressed as mean±SEM. Mann-Whitney non-parametric U-test was used to compare the number of VEBs and the duration of VT and VF among the groups. Fisher-Irwin exact test (with Yates correction) was used to analyze the occurrence percentage of VT and VF. Statistical analysis of the area at risk, volume and percentage of infarct size was performed between the control and test groups by one-way ANOVA and LSD post test. The values of P<0.05 were considered significant.

## Results

**Figure 1:**
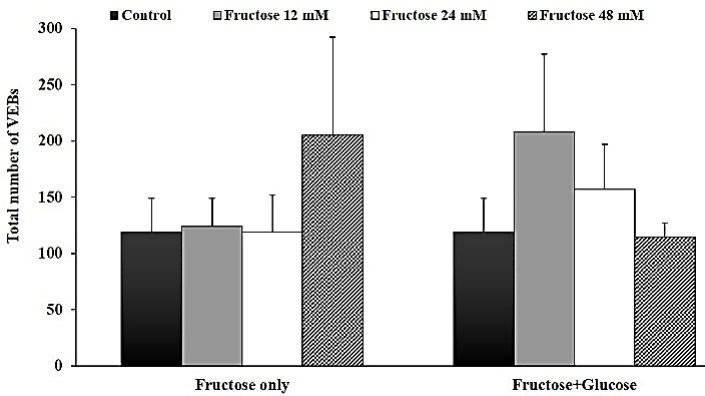
Effects of Krebs-Henseleit solution containing concentrations of 12, 24 and 48 mM fructose instead of glucose, as well as normal Krebs (having glucose) with 12, 24 and 48 mM fructose on the total number of VEBs during the first 30 min of reperfusion in isolated rat hearts. All data is expressed as mean±SEM. Number of rats in each group is equal to 7-10.

**Figure 2:**
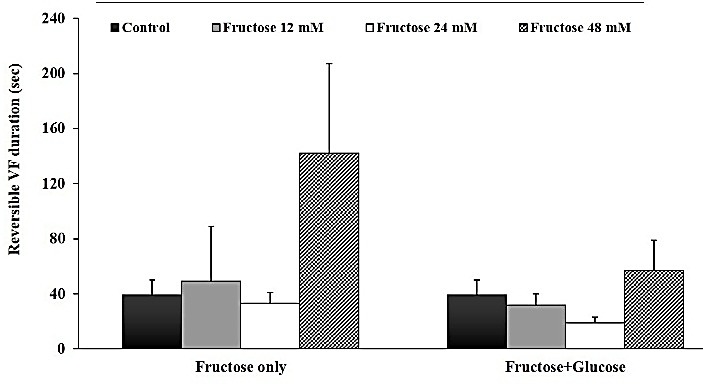
Effects of Krebs-Henseleit solution containing concentrations of 12, 24 and 48 mM fructose instead of glucose, as well as normal Krebs (having glucose) with 12, 24 and 48 mM fructose on the Rev VF time (second) during the first 30 min of reperfusion in isolated rat hearts. All data is expressed as mean±SEM. Number of rats in each group is equal to 7-10.

Effects of post-ischemic administration of fructose on reperfusion-induced cardiac arrhythmias after 30 min zero flow global ischemia are summarized in Table 1. Perfusion of glucose-free K/H solution in which glucose was replaced with 12, 24 and 48 mM of fructose, had no statistically significant inhibitory effect on the total number of VEBs compared to the control group. Similarly, administration of normal K/H solution containing glucose plus 12, 24 and 48 mM of fructose did not produce significant reduction in the number of VEBs (Figure 1). In addition, statistical comparison did not show any significant difference between the control and all treatment groups on Rev VF duration in both sets of experiments (Figure 2).

**Table 1:** Effects of post-ischemic administration of fructose on reperfusion-induced cardiac arrhythmias after 30 min zero flow global ischemia in isolated rat hearts. Data are represented as mean±SEM. N=7-10 in each group. VT: Ventricular Tachycardia, VEBs: Ventricular Ectopic Beats (Single+Salvos+VT), Rev VF: Reversible Ventricular Fibrillation, Irrev VF: Irreversible Ventricular Fibrillation, K/H solution: Krebs-Henseleit solution.

**Groups**		**Reperfusion arrhythmias**						
		**VEBs** **number**	**VT** **number**	**VT** **duration (sec)**	**Rev VF** **duration (sec)**	**Rev VF** **incidence (%)**	**Irrev VF** **incidence (%)**	**VT** **incidence (%)**
**Control**	**Group 1**	119±30	0	0	39±11	0	0	0
**Fructose without normal glucose in K/H solution**	**Group 2 (12 mM)**	124±25	7±7	1.25±0.5	49±40	50	0	13
	**Group 3 (24 mM)**	119±33	2±2	0.25±0.25	33±8	13	0	16
	**Group 4 (48 mM)**	205±87	35±25	5.75±3	142±65	50	0	38
**Fructose with normal glucose in K/H solution**	**Group 5 (12 mM)**	208±69	34±12	4.28±3	32±8	20	0	43
	**Group 6 (24 mM)**	157±40	16±5	2.5±1	19±4	50	0	38
	**Group 7 (48 mM)**	114±13	10±3	3±2	57±22	55	0	25

**Figure 3:**
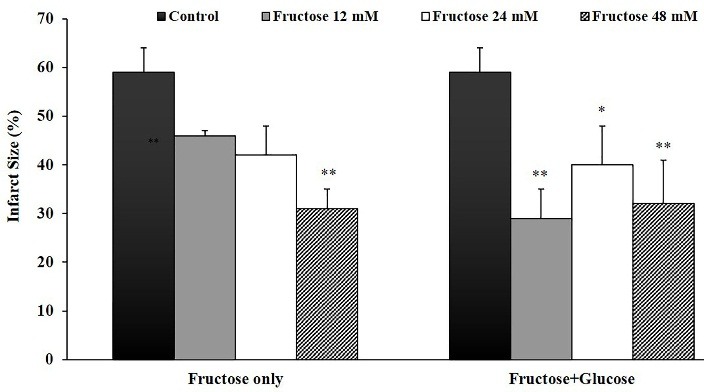
Myocardial infarct size in the control and treated groups receiving 12, 24 and 48 mM of fructose with/without glucose in Krebs-Henseleit solution for 20 min at the beginning of 120 min reperfusion. Data are represented as mean±SEM. **P<0.01, *P<0.05 versus the control group. N=7-10 rats in each group.

Effects of fructose on the infarcted volume and percentage of infarct size in the isolated rat hearts are summarized in Table 2. Perfusion of K/H solution containing different concentrations of fructose with or without glucose for 30 min after global ischemia markedly reduced infracted volume in the all treatment groups in comparison with the control group. The effect was especially significant in the hearts that were perfused with fructose and glucose co-administration. In parallel to the above results, percentage of infarct size was clearly reduced by post-ischemic administration of fructose in the all treatment groups (Figure 3). Based on the infarct size value in control group (59±5%), percentage of infarct size reduction were 22 % (in group 2, P>0.05), 29 % (group 3, P>0.05), 47.5 % (group 4, P<0.01), 51 % (group 5, P<0.01), 32 % (group 6, P<0.05) and 46 % (group 7, P<0.01), respectively. There was no significant difference between the treatment groups in infarcted volume and percentage of infarct size.

**Table 2:** Effects of administration of fructose on myocardial infarcted volume and infarct size after 30 min zero flow global ischemia followed by 120 min reperfusion in isolated rat hearts. Data are represented as mean±SEM. **P<0.01, *P<0.05 versus the control group using one way ANOVA with LSD post test. N=7-10 rats in each group. K/H solution: Krebs-Henseleit solution.

**Groups**		**Area at risk (mm)³**	**Infarcted volume (mm)³**	**Infarct size (%)**
Control	Group 1	140±5	82±7	59±5
Fructose without normal glucose in K/H solution	Group 2 (12 mM)	130±9	65±3	46±1
	Group 3 (24 mM)	140±8	60±13	42±6
	Group 4 (48 mM)	131±6	42±6**	31±4**
Fructose with normal glucose in K/H solution	Group 5 (12 mM)	144±8	42±9**	29±6**
	Group 6 (24 mM)	143±5	55±8*	40±8*
	Group 7 (48 mM)	125±9	45±13**	32±9**

## Discussion

Coronary heart disease is estimated to be the leading cause of death worldwide by 2030.^32^ In spite of advances in treating ischemic heart diseases in the past three decades, acute MI is a major cause of death in developed countries.^33^ Reperfusion (restoration of blood flow to ischemic tissue) after an ischemic period can be more fatal than ischemia itself due to production of destructive free radicals and calcium overloading, etc.^34^

In the current study, we focused on the effects of fructose as a potential postconditioning agent against I/R-induced arrhythmias and infarct size in isolated rat heart. In the first set of experiments, glucose in three groups of isolated hearts was removed from K/H solution and 12, 24 and 48 mM of fructose were replaced. In three other groups, the hearts were perfused with K/H solution containing the above concentrations of fructose in the presence of glucose. The results showed that replacement of glucose by fructose in K/H solution did not have a significant effect on decreasing the number, duration, or occurrence of dangerous reperfusion arrhythmias such as VT and VF. Similarly, the consumption of fructose with normal glucose in K/H solution did not produce significant inhibitory effect against reperfusion arrhythmias. However, fructose alone reduced the size of infarction area and percentage of infarct size in all concentrations used. This effect was statistically significant at a concentration of 48 mM (group 4). In addition, co-administration of fructose with normal glucose in K/H solution (groups 5, 6, and 7) significantly reduced the infarction volume and infarct size with all the prescribed concentrations. In consistent with these findings, results of our previous study demonstrated that pre-ischemic administration of fructose during 30 min stabilization period followed by 120 min reperfusion reduced the infarct size in isolated hearts. The difference is that the effects of fructose on the reduction of arrhythmias were significant in the previous study.^24^ The effect of fructose on the reduction of arrhythmias seems to be due to its pre-ischemic administration and is related to the protective role of preconditioning phenomenon.

Long-term feeding with fructose can be harmful to the myocardium, because the heart is a sensitive tissue to insulin.^10^ Feeding rats with high levels of fructose causes hyperinsulinism, which increases the blood pressure.^11^ Unlike fructose-induced hypertension in rodents, acute fructose administration in humans did not have an effect on sympathetic system activity and hypertension.^15^ Studies on rats have also shown that fructose causes endothelial dysfunction due to inhibition of nitric oxide synthesis,^18^ fibrosis around coronary arteries,^16^ dysfunction of end arterioles in skeletal muscles,^17^ and stimulating the production of free radicals.^19^ In the study conducted by Jordan et al., a group of rats were fed with fructose for 4 weeks and the other groups received it for 3 days. Only the first group showed insulin resistance syndrome, but the decrease in the size of infarction area was observed in both groups. In this study, phenomenon of preconditioning is introduced as the cause of protective effect of fructose and it is not associated with metabolic changes caused by long-term fructose diet.^23^ In the study carried out by Joyeux-Faure et al., a diet containing high fructose was given to rats for 4 weeks, in a way that 58% of total carbohydrate was fructose. The results of their study indicated that occurrence of VF and VT during ischemia as well as reperfusion between control and fructose groups was not significantly different, but there was a decrease in the size of infarcted area. In that study, increased plasma levels of vitamin E (an antioxidant) was introduced as the main reason for protective effects of fructose.^35^ Similar to the results of Joyeux-Faure et al. in chronic fructose feeding, we found that acute administration of fructose reduced infarct size without any significant effect against I/R-induced arrhythmias.

Cardiac cell capacity for fructose uptake has not been reported, however since GLuT5 has a low tendency to glucose, this compound is likely to act as a good functional carrier for fructose in the heart.^10^ Although fatty acid oxidation is the main source of ATP for cardiomyocytes, there is some evidence that the ATP produced by glycolysis plays an important role in calcium transmission. The close relationship between glycolytic enzymes and calcium carriers reminds us that the stimulation-contraction coupling process specific to the heart is essentially dependent on the ATP produced by glycolytic. The ATP produced by fructose during glycolysis can provide a stimulation-contraction coupling process by Ca^2+^ carrier, but the direct role of fructose in the performance of cardiomyocytes is not clearly stated.^10^ In three treatment groups of the present study that evaluated effect of fructose alone, the results indicate that fructose can cause cardiac cells contraction in the absence of glucose, although these contractions are not as advanced as with glucose utilization state. As mentioned before, since the GLuT5 carrier has a slight affinity to glucose, there is no competition in the entry of these two monosaccharides into cardiac cells, and fructose easily enters the cells by the carrier.^10^ Considering that fructose brings a shortcut to the main step of glycolysis regulation (conversion of glucose 6-phosphate to fructose 1,6 bisphosphate by phosphofructokinase), when glucose metabolism is inhibited by phosphofructokinase or when there is no enough glucose, fructose can continue to enter the glycolysis pathway. This situation leads to more energy production for cardiac cells.^4^ Therefore, it can be deduced that since one of the injuries caused by I/R is cell necrosis and loss of cell life, fructose will probably prevent the accumulation of Ca^2+^ and Na^+^ within cardiac cells by providing ATP, then preventing cell necrosis. This hypothesis is consistent with the result of the present study. So that, in the groups that glucose was co-administered with 12, 24, and 48 mM of fructose, it was observed that the infarction volume and infarct size were significantly decreased compared to the control group. Future studies in this field could further clarify the effects of fructose in cardiac I/R conditions.

## Conclusion

In general, findings of this study showed that administration of high concentrations of fructose alone or together with normal glucose, reduces the volume and percentage of infarct size in isolated hearts after global ischemia. However, it does not have a significant inhibitory effect against reperfusion-induced dangerous cardiac arrhythmias such as VF and VT. Probably, fructose prevents necrosis and death of cardiomyocytes by supplying adequate ATP for cardiac functions during I/R, and then reduces infarct size.

## Acknowledgments

The present work was supported by the Research Affairs of Tabriz University of Medical Sciences, Tabriz, Iran. This article is based on a thesis submitted for Pharm D degree (No.3671) in Faculty of Pharmacy, Tabriz University of Medical Sciences.

## Ethical Issues

All the experiments were carried out under ethical guidelines of Tabriz University of Medical Sciences, for the care and use of laboratory animals (National Institutes of Health Publication No 85-23, revised 1985).

## Conflict of Interest

The authors report no conflicts of interest.
